# Setting up a Paediatric Rapid Access Outpatient Unit: Views of general practice teams

**DOI:** 10.1186/1471-2296-9-54

**Published:** 2008-09-29

**Authors:** Lisa Williams, Jane Fryer, Rachel Andrew, Colin Powell, Jim Pink, Glyn Elwyn

**Affiliations:** 1 Department of Primary Care & Public Health, School of Medicine, Cardiff University, 3rd Floor Neuadd Meirionnydd, Heath Park, Cardiff, CF14 4XN, UK; 2 Department of Paediatrics, School of Medicine, Cardiff University, The Children's Hospital for Wales, Heath Park, Cardiff, CF14 4XW, UK

## Abstract

**Background:**

Rapid Access Outpatient Units (RAOUs) have been suggested as an alternative to hospital inpatient units for the management of some acutely unwell children. These units can provide ambulatory care, delivered close to home, and may prevent unnecessary hospital admission. There are no qualitative data on the views of primary care practitioners regarding these types of facilities. The aim of the study was to explore the opinions of primary care practitioners regarding a newly established RAOU.

**Methods:**

The RAOU was established locally at a district general hospital when inpatient beds were closed and moved to an inpatient centre, based six miles away at the tertiary teaching hospital.

Qualitative, practice based group interviews with primary care practitioners (general practitioners (GPs), nurse practitioners and practice nurses) on their experiences of the RAOU. The data collection consisted of three practice based interviews with 14 participants. The interviews were recorded and transcribed verbatim. Thematic content analysis was used to evaluate the data.

**Results:**

There was positive feedback regarding ease of telephone access for referral, location, and the value of a service staffed by senior doctors where children could be observed, investigated and discharged quickly. There was confusion regarding the referral criteria for the assessment unit and where to send certain children. A majority of the practitioners felt the utility of the RAOU was restricted by its opening hours. Most participants felt they lacked sufficient information regarding the remit and facilities of the unit and this led to some uneasiness regarding safety and long term sustainability.

**Conclusion:**

Practitioners considered that the RAOU offered a rapid senior opinion, flexible short term observation, quick access to investigations and was more convenient for patients. There were concerns regarding opening hours, safety of patients and lack of information about the unit's facilities. There was confusion about which children should be sent to the unit. This study raises questions regarding policy in regard to the organisation of paediatric services. It highlights that when establishing alternative services to local inpatient units, continual communication and engagement of primary care is essential if the units are to function effectively.

## Background

There is considerable debate about how best to organise the provision and delivery of urgent and emergency care for acutely unwell children.[1] The National Service Framework emphasises that the delivery of such care should be '*as close to home as possible while ensuring that their care is of the highest quality'*.[2] Some smaller inpatient units have had to close due to staffing pressures, the European working time directive and an inability to maintain a fully trained workforce.[3]

Paediatric emergency assessment units, acute observation units, acute assessment clinics, rapid access outpatient units (RAOUs) or satellite units away from the inpatient departments are possible alternatives to a local inpatient unit.[4] For consistency the abbreviation RAOU will be used encompassing the different terms used in the literature.

Studies examining the impact, cost effectiveness, safety and effect on hospital admissions are few but generally supportive of such developments.[4] As the pressure increases to reconfigure local services it is an imperative that there is ongoing evaluation of their role.

There has been little exploration of views from Primary Care of these alternatives to hospital inpatient units.[5] The only study identified is a postal questionnaire of the views of GPs in Northern Ireland on a RAOU set up in 1996. The sample size was small with 37 responses out of 57, equating to a response rate of 65% with possibly some bias in the results. The study revealed that 97% of GP's found the RAOU easy to access, 94% felt that feedback was appropriate and 61% of GPs wanted to see the opening hours extended.[6]

In Cardiff and Vale NHS Trust a 20 bedded paediatric inpatient unit (Llandough Hospital) was closed and the facilities moved six miles away in Cardiff to the new Children's Hospital for Wales, a tertiary teaching hospital. A RAOU was established on the site of the old inpatient unit in order to deliver a local service for the families and general practitioners. The aim of this study was to explore primary cares views on the service, two years after it was established.

## Methods

This study was approved by the South East Wales local research ethics committee. The study population were primary care practitioners working in Cardiff and the Vale of Glamorgan, who had been able to access the RAOU for the past 2 years.

### The Rapid Access Outpatient Unit

A satellite RAOU was established at Llandough Hospital's Children's Centre when the inpatient wards were closed in March 2005. The unit is currently open from 9-5 pm Monday to Friday, excluding Bank Holidays. The last referral is accepted at 4 pm [w1]. It is staffed by two experienced paediatric nurses, one specialist registrar in paediatrics and a consultant paediatrician in the afternoon. This unit has access to radiology and laboratory services. There is no emergency department in the hospital, no paediatric intensive care unit, but there is an acute anaesthetic team who could assist if a child were to require airway stabilisation. All the paediatric, medical and nursing staff are trained in advanced paediatric life support.

The Children's Hospital for Wales provides the local secondary care service to Cardiff and the Vale of Glamorgan. It sees 5000 acutely unwell children per year in the Children's Assessment Unit, 25,000 children in the Emergency Unit and also provides tertiary services for children in Wales. The hospital has inpatient beds, a paediatric high dependency unit, a paediatric intensive care and neonatal intensive care unit.

In its first two years the RAOU has seen 3000 children referred from local primary care practitioners. Practitioners refer directly by telephone speaking to a paediatrician or experienced nurse, when a decision is made whether the child should be seen in the unit or sent directly to the Children's Hospital for Wales (known locally as "The Heath"). At its inception, all surgeries were informed of the new unit and referral criteria by the Local Health Board. Since then a number of reminders, flyers and emails have been sent on a regular basis. The characteristics of patients seen in the RAOU to date are to be described in a further paper.

### Study Design

The decision was taken to arrange practice based interviews, as we felt this would allow practitioners to discuss their experiences and respond to each others views in a matter which would not be possible in one to one interviews [w1]. Practices with less than three practitioners were therefore excluded as it was felt that in these the group interviews would be too small for the desired group discussion. The lead GPs of all practices in the Cardiff and Vale of Glamorgan area with three or more clinicians and access to the RAOU were sent information on the study and invited to participate. Follow up contact was then made with the practice managers. Each interview was facilitated by two academic GPs (LW & JF or JP) [w1].

The interviewers used four key topic areas to guide the session and to ensure important areas were covered (Table 1). However, free discussion was encouraged. The discussion was digitally recorded and transcribed verbatim. The transcriptions were then analysed using thematic content analysis. Analysis began with open coding describing each section within the transcripts. Using comparison across the transcripts, the open codes were refined into major themes which provided a coding frame for analysis. Initial coding was carried out by LW with thematic analysis conducted by both LW and JP to reach as consensus [w1].

**Table 1:** The topic areas covered in group interviews

1	Understanding of RAOU services
2	Personal experiences and views of RAOU
3	Factors that are valued about the current RAOU service
4	Views of how the RAOU service should develop

RAOU = rapid access outpatient unit

## Results

Recruitment was difficult, contacting the lead GPs in 25 practices by letter led to no initial responses. Following contact with practice managers, four practices provisionally agreed. One subsequently was unable to offer a date for the interview, despite repeated contacts. Many other practices promised a response but few were received. Of the nine refusals received, most related to lack of time or participation in other research projects.

Three practice-based interviews were conducted with a total of 14 primary health care professionals. The first practice interview involved two GPs, a GP (in training), a nurse practitioner and two nurses. The second interview involved five GPs and the third practice interview involved three GPs. Of the 14 participants four used the RAOU regularly, six used it less frequently. None of the nursing professionals were aware of the RAOU. We were able to describe four themes, which are summarised in Table 2.

**Table 2:** The main themes

**1. RAOU benefits**: good telephone access, observation, access to specialist opinion, diagnostic tests and location.
**2. Referral difficulties**: issues regarding referral criteria, access restriction, and mixed messages from units regarding each others criteria. Does more choice equal more complexity?
**3. Lack of information**: limited understanding and awareness of unit – the exact remit and the nature of facilities available.
**4. Infrastructure and safety**: concern expressed regarding facilities, patient safety and whether the RAOU had long-term sustainability.

RAOU = rapid access outpatient unit

### Theme 1: RAOU Benefits

Most of the participants felt the RAOU was a useful additional service and saw it as a good "halfway house". The ability to offer specialist opinion locally, with facilities to observe children and access rapid diagnostic tests was highly valued.

There was excellent feedback regarding the ease of telephone access to the unit. Practitioners valued the quick access and the call handling by nurses, particularly as this avoided the wait to be connected to the on call doctor via switchboard. A number of the participants commented on how helpful the staff were in comparison to referrals to other hospital departments.

*They're always really friendly... They'll see the child straight away so it's easy, there are never any sort of hard questions, they just want to know the basics ... So yes the ease of access is good*.

GP (in training) in Practice 1

Practitioners valued the unit's ability to observe children, which often allowed safe discharge home once assessment had taken place. This meant that children who previously may have been admitted for a short stay could be observed and discharged. They saw this period of observation as very useful for a range of conditions including gastroenteritis, bronchiolitis, asthma and upper respiratory tract infections.

*Kids vary. They may be bad in the morning, good in the afternoon: give them Calpol and they may be well an hour later, so I think its nice just having somewhere to send them in to be observed. You know ... if mum says he really hasn't taken any fluids, you don't always believe her, but it's nice just to know they can be watched for a few hours*.

GP (in training) in Practice 1

The ability of the unit to provide a second opinion by a senior specialist was also valued, Several practitioners commented that this was reassuring not just for parents but also for themselves.

*I've found it really useful for the 'rule of threes' consultations. A patient comes back for the third consultation for the same illness and it either means that your diagnosis is wrong, they've lost confidence in you or they're very anxious parents. In any of those three scenarios then it's really useful to have another opinion*.

GP 1 in Practice 2

Practitioners valued the fact that the unit could quickly access diagnostic tests, such as urine testing. They commented that often if these diagnostic tests were negative, an admission could be prevented.

*Sometimes ...you're sending him up purely for an chest x-ray, if that prevents a hospital admission then its all for the good*.

GP 1 in Practice 2

Practitioners commented that location was important for the patients. This was particularly so for families with poorer access to transport.

*Because it is a long way to go to the Heath from here... I mean our practice is mainly social class V/VI ...A lot of the young mothers don't have a lot of money, they don't have access to regular transport and so having a paediatric assessment unit that is closer ... is handy actually*.

GP 3 in Practice 3

### Theme 2: Referral difficulties

The most striking theme was uncertainty regarding the referral of patients, particularly the interface between the two local paediatric units. There was confusion about where to send children and the exact referral criteria for the RAOU. Some practitioners had experiences of being 'bounced' between centres when trying to refer a child. The practitioners also described experiences of differences of opinion as to whether a child should be referred to the inpatient unit or be seen in the RAOU when speaking to the RAOU staff. This created delays and unwelcome stress, and some practitioners worried it may sway their original decision about the safest and most appropriate place for the child to be reviewed [w1].

That was my worry... where you sort of made your decision but you thought they probably ought to go to the Heath and then you speak to somebody at the Heath they say "oh you know we're really busy here, have you thought of sending them to Llandough"

GP 2 in Practice 2

*What I thought would have been a good admission for the Heath but I was told it was better for Llandough and Llandough disagreed and I was caught in that horrible position... "I don't know where to send you"*.

GP 2 in Practice 3

There was concern about the restrictions caused by limited opening hours. The majority of practitioners felt that the final referral time of four o'clock was too early, as one hour for observing the unwell child may be inadequate to formulate an appropriate management plan [w1].

*The dehydrated ones... if it comes to about four o'clock then it's too late ... You need those children to be there quite early, for there to be enough time*... [for assessment]

GP 3 in Practice 2

*You quite often get mums coming in from work and seeing a sick child and coming in at half past five quarter to six, so it would be nice if they were open longer*.

Nurse Practitioner Practice 1

### Theme 3: Lack of Information

There was considerable concern regarding the lack of information about the RAOU. Although referral criteria had been sent to surgeries when the unit opened, not all referring practitioners had received them and many were vague about the unit's details..

*I've got it somewhere, in my rainforest worth of protocols and guidelines in my room*.

GP 1 in Practice 3

Practitioners all wanted more information regarding the unit and its facilities. Common queries were regarding the number of available beds, the staffing arrangements, and the availability of investigations..

Do they do investigations with bloods and things like that there?

GP 2 in Practice 1

For some practitioners the lack of information prevented them using the service, particularly as they were much more aware of the facilities available at the inpatient unit.

*It's easy to just ring the Heath and say "please see this child, yes I think they need admission", because I don't know, but I know the Heath has got all resources*.

GP 1 in Practice 1

### Theme 4: Infrastructure and safety

There were some worries regarding the safety of the unit, due to it not being an inpatient centre with all the associated facilities. There was concern that children could deteriorate quickly between being triaged by the GP as fit for the RAOU and arrival on the unit, which it was feared would be less able to provide emergency treatment of very unwell children. These fears were compounded by a lack of knowledge of the facilities available. However, others thought these concerns were counter-balanced by the benefits of the service and the understanding that an ill child at the RAOU would be managed and then transferred.

I think another difficult thing is knowing that the unit at Llandough has been shrunk down so much and all the acute stuff is now at the Heath. Sending acutely ill children to Llandough feels a bit uncertain, in case they then have to be transferred to the Heath

GP 1 in Practice 1

There was discussion surrounding the sustainability of the service. Practitioners were wary of the risk of closure of the unit due to other acute services being moved to the teaching hospital.

## Discussion

This Rapid Access Outpatient Unit offers flexible short term observation, quick access to investigations and is more convenient for patients. However, because it is located at a distance from the main paediatric inpatient unit and only open 9 am to 5 pm five days a week, concerns exist amongst GPs and their teams. There is confusion about which children and when to refer to such units and there are worries about the level of infrastructure and staffing support to ensure patient safety.

The practice based interviews demonstrate that primary care practitioners have a range of views on the services offered. There was almost universal agreement on the ease of access by telephone. All participants valued the senior doctor led service, as well as the ability to observe children and give a second opinion. The concerns regarding the unit seem to stem from a lack of clarity on referral pathways and a lack of information. Practitioners often felt stuck in the middle, with the two units disagreeing on the appropriate referral unit. Clearly, making a decision on whether a child needs admission is challenging and such disagreement can complicate referrals. This was compounded by a lack of knowledge of the facilities of the unit and what it could offer, which also raised worries regarding safety. Lastly, some practitioners were unsure of the RAOU's longer term sustainability.

The weakness of this study is the small sample size which could mean that only limited perspectives were gained. However, even with three interviews, the team was reassured that the same themes came up repeatedly. Recruiting practices for group interviews is not easy due to the practical problem of getting a group together when all the individuals are busy. The possibility of telephone interviews was discussed as another method for recruitment especially as this would have enabled purposive sampling. Each method has the practical problem of trying to sample busy clinicians either singly or as a group. We felt there that using the practice meetings for the sampling would be easier. The academic GPs interviewers could have influenced the focus groups by their presence but attempted to interfere minimally with the discussions. There was minimal contradictory data and indeed the same themes came up repeatedly in all the three focus group discussion

The strength of the study is that we were able to get in-depth views of the participants in a realistic setting. We believe bringing together the practices' clinicians in a familiar group setting resulted in participants being more comfortable and likely to share their views than if they had been with clinicians from other practices. Our methods could be replicated elsewhere in the UK and in this respect we believe our method and analysis was appropriate.

Although there has been research evaluating GPs' views of RAOUs using quantitative methods (questionnaires) [6], we believe this is the first qualitative research into primary care practitioners' views of a RAOU. Using qualitative methods this study reveals some of the more complex issues regarding a RAOU. The concerns raised by the participants underline the difficulties of running such a service, despite the benefits also discussed. A local senior led service is valued by clinicians and patients alike, but currently the RAOU is limited by its referral criteria and its opening hours. This creates tensions as practitioners can sometimes get caught in between two referral centres. A local service may lack the facilities of a larger paediatric centre and this leads to concern about safety as well as long term sustainability. We believe this study highlights the vital importance of early and repeated communication between all stakeholders when such a service is established. Practitioners need to have clear information on the role of the service, its referral criteria, opening hours and facilities. Without this information practitioners may lack confidence and use alternative services.

This study appears to confirm the findings of a study in Northern Ireland which revealed that 61% of GPs wanted to see the opening hours extended (unit open Monday-Friday 9-5 pm).[6] The importance and relevance of opening hours can be demonstrated by research that has shown that about 50% of paediatric admissions are in the evening and at night.[7]

This study raises questions in regard to the organisation of paediatric services. The Royal College of Paediatrics and Child Health have suggested RAOUs as an example of ambulatory care which can prevent inappropriate admissions.[8] A large number of studies have been able to demonstrate a reduction in admissions [9-12] but few studies have considered the wider implications for the service itself and primary care.

This study has highlighted some areas of concern regarding referral, access restrictions and the potential safety of the units. These concerns should be considered in the future planning of service delivery. One key consideration is to provide a suitable interface for primary care practitioners to carry out referral triage, and one access point (i.e. a single telephone number) leading to tiered care may be a good solution. Our results emphasize the need for further studies to look at RAOUs' future role in paediatric emergency services. They also demonstrate that it is essential for primary care to be engaged right at the beginning of the establishment of these units to allow information sharing. Continual communication with local GP practices will also allow a more effective use of such facilities.

## Competing interests

CP is involved in planning, commissioning and managing the RAOU.

## Authors' contributions

LW participated in the design of the study and organised, co-ordinated and facilitated the focus groups in primary care. LW also contributed to study analysis involving initial coding and thematic analysis and writing the paper. JP participated in the design of the study and organised, co-ordinated and facilitated the focus groups in primary care and contributed to the thematic analysis and writing the paper. RA participated in the design of the study and organised, co-ordinated and facilitated the focus groups in primary care and writing the paper. JF participated in the initial concept and design of the study, co-ordination and facilitation of the focus groups and contributed to the final paper. CP conceived of the study, participated in study design and coordination and advised on the analysis and final paper. GE participated in study design and coordination and advised on the qualitative analysis and writing the final paper. All authors read and approved the final manuscript.

What is already known on this topic?

Rapid access outpatient units have been suggested as an alternative to inpatient units for the management of acutely unwell children.

Many studies have been able to demonstrate a reduction in admissions with the introduction of emergency assessment units.

There is very little research on primary care opinions and experiences of these new assessment units.

What this study adds

Practitioners felt that the rapid access outpatient unit (RAOU) offered a rapid senior opinion, with flexible short term observation, quick access to investigations and a more convenient service for patients.

Primary care practitioners were concerned regarding referral problems, opening hours, potential safety issues and a lack of information regarding the unit.

Further research is required to consider the future role of RAOUs in paediatric emergency services.

## Pre-publication history

The pre-publication history for this paper can be accessed here:


